# Cultural Norm Transmission/Disruption amongst Somali Refugee Women: The Beauty and Privilege of Intergenerational Relationships

**DOI:** 10.3390/socsci13080432

**Published:** 2024-08-21

**Authors:** Zamzam Dini, Cawo Abdi, Beatrice (Bean) E. Robinson, Jennifer Jo Connor

**Affiliations:** 1 Department of Family Social Science, University of Minnesota, 1985 Buford Ave., St. Paul, MN 55108, USA;; 2 Department of Sociology, University of Minnesota, 909 Social Sciences Building, 267 19th Ave. S, Minneapolis, MN 55455, USA;; 3 Eli Coleman Institute of Sexual and Gender Health, Department of Family Medicine and Community Health, University of Minnesota, 1300 S 2nd St., Suite 180, Minneapolis, MN 55455, USA;

**Keywords:** intergenerational relationships, Somali women empowerment, FGC and decision-making, refugee family systems, social movements

## Abstract

Since the onset of the Somali civil war in the late 1980s, more than 2 million Somalis have been internally displaced or crossed international borders to seek haven. Yet, research on diasporic Somali women’s intergenerational communication about marriage, sex, and female genital cutting (FGC) remains scant. This paper draws from data we collected from 15 women over the age of 45 who were part of a much larger project on refugee women and sexual health and well-being. The analysis centers on how Somali women across the generations recalibrate definitions of family. We analyze the new roles that sisters, aunts, and grandmothers occupy in the lives of younger women, as family dispersal often results in the absence of biological mothers. In the new settlement, the findings showcase both continuity and change in how sex, marriage, and female genital cutting (FGC) are discussed among female family members. Our findings support not only the dynamic nature of family roles that women occupy across generations but also the malleability of cultural practices as families navigate changing cultural, legal, and social norms in their new settlements.

## 1. Introduction

Migration can be synonymous with change, as those who settle in new places often encounter forces that create potential opportunities and stressors to reshape their world-views. These changes are often represented within the debates in migration studies and the social sciences, including acculturation, assimilation, and multiculturalism (Berry 2003, 2006; Brosseau and Dewing 2018; Mathieu 2018; Rumbaut 1997; Waters and Jiménez 2005). Such key concepts anchor within them the multiple ideas, practices, and possibilities in a migrant or refugee group’s (non)choices in embracing, rejecting, or appropriating dominant cultural practices and ways of being and living life in new settlements/homes (Castles 2002; Georges 1990; Hondagneu-Sotelo 2003; Hondagneu-Sotelo and Messner 2013; Kibria 1995). Recent debates around transnationalism also underscore the need to move away from “narrow” identities and modes of belonging that some of the earlier concepts might represent. Unlike earlier scholarship, which centered assimilation as a one-way mode of migrant incorporation, with newcomers embracing the American mainstream way of life, newer scholarship pushes us to appreciate and recognize the multiplicity of political, economic, and cultural belongings migrants often navigate and create for themselves. Their families and their communities have complex, multifaceted, and multi-sited lives that transcend borders and limited nation-centric identities (Castles 2002; Mahler 1998; Schiller et al. 1992).

While changes and continuities are two sides of the same coin for all groups, whether native or newcomer, migration renders such micro and macro forces more overt. As newcomers struggle to both respect and continue highly entrenched cultural practices brought from home while at the same time, settlement can render these practices questionable and, at times, even criminal. These contestations might emerge from extremely conflicting meanings into bodily autonomy, for example, or what constitutes family, acceptable sexual norms, womanhood, and beyond in diverse regions of the world and in diverse societies.

### Female Genital Cutting

One example that concretizes such contestations about women’s bodies, sexualities, and womanhood is that experienced by migrants and refugees coming from regions where FGC (at times referenced as female genital mutilation) is prevalent (Johansen 2002; Johnson-Agbakwu and Manin 2021; Pastor-Bravo et al. 2021). FGC is common in Somalia, with an estimated rate of 98%, making FGC a social norm (Barrett et al. 2021; UNICEF 2020). In a recent meta-analysis about FGC prevalence, girls from regions that practice FGC were 13.71 times more likely to have undergone FGC if their mothers and grandmothers had also been cut (Ayenew et al. 2024). The World Health Organization (2008) has identified several types of FGC; however, previous research has demonstrated that for Somali women, FGC is typically conceptualized as Sunna (in WHO language, types I, II, and IV) and Pharonic (also known as type III or infibulation) (Johnson-Agbakwu et al. 2023). The majority of Somali women born in East Africa are infibulated and hence have experienced the most extreme form of FGC (Barrett et al. 2021). Type III involves “the narrowing of the vaginal introitus with the creation of an external vulvar covering by fusion of either the labia minora or labia majora with or without excision of the external clitoral glans” (World Health Organization 2008, p. 4). Other forms of FGC may involve pricking the genitals (type IV), removal of clitoral tissue (type I), or removal of clitoral tissue and labia minora, and possibly labia majora (type II) (World Health Organization 2008). While there are no known health benefits, FGC is associated with an increased risk of acute complications, such as infection, shock, or bleeding (Berg et al. 2014). Long term complications also exist as FGC leads to obstetric and neonatal complications, urinary problems, and mental health and sleep problems throughout the life course (Abdalla and Galea 2019; Andro et al. 2014; Berg et al. 2014; Bertuit et al. 2023; Kulaksiz et al. 2022).

The impact of FGC on sexuality has been explored in both qualitative and quantitative studies. Meta-analyses indicate that FGC is associated with lower sexual function across several domains, including desire, arousal, lubrication, orgasm, satisfaction, and pain (Berg and Denison 2012; Nzinga et al. 2021; Pérez-López et al. 2020). However, findings varied across studies, which may be attributed to the type of FGC women have been subjected to. For example, sexual pain tends to be higher in women with type III as opposed to types I and II (Nzinga et al. 2021). Additionally, not all women who have experienced FGC report sexual problems, highlighting the heterogeneity in outcomes (Johnsdotter 2018).

Qualitative research can elucidate variations in outcomes. Participants in our qualitative research in a sample of Somali women in the U.S. under age 45 provided descriptions of different sexual health trajectories (Connor et al. 2023). Most experienced extreme pain when first sexually active, accompanied by low or no sexual pleasure. However, there was not a singular story. Participants’ stories varied in whether or not their spouse helped them cope with pain, they sought medical assistance, they used lubrication, and they engaged in painful sex regularly. Participants also differed on whether or not they began to experience sexual pleasure and/or a reduction in sexual pain as time passed. Throughout the stories, the importance of spousal relationships, communication, and female agency were key to mitigating pain. Notably absent was sex education, either formalized or informal from elder family members. However, many participants shared that they sought advice from female elders on deinfibulation (i.e., medical procedure to open the narrowed introitus) (Connor et al. 2023, 2024). This finding explains why this paper draws from older women’s narratives about sexual communications, as they were more likely to discuss this topic compared to younger women.

Migration can interrupt normalization of FGC and its possible complications. Settlement in countries where FGC has been illegal for years demands newcomers to rethink older practices because of local laws that criminalize such practices (Barrett et al. 2021). It is possible that these laws are not the only force potentially producing cultural shifts; however, such changes might also be due to individual or communal attitudinal shifts in foundational ideas that justified these practices themselves in the home country. The latter changes can be driven by the reinterpretation of culture and/or religion or the discovery of new logic that these practices were not universal as previously imagined, were unnecessary, or even not enshrined in faith as many might have been convinced in their home countries (Johnsdotter and Essén 2016; Salah et al. 2024).

In essence, we must acknowledge that the root of shifts in cultural practices is complex, and it might be hard to pinpoint the exact causes. Both sociological and psychological studies acknowledge the complex individual and family processes of migrant and refugee incorporation into new settlements. As John W. Berry’s acculturation theory pioneered, “the long-term consequences of this process of acculturation are variable, depending on social and personal variables that reside in the society of origin, the society of settlement and phenomena that both exist before and arise during, the course of acculturation” (Berry 1997, p. 5). There is also recognition of the varying levels of change that might emerge with variations across sex, gender, age, and migration timeline, all factors potentially shaping shifts in cultural attitudes and practices (Abdi 2007; Furnham and Bochner 1986).

Family and communication about all facets of life are also intrinsically linked. Communication often has multiple complex and even contradictory forms, and both verbal and non-verbal connections and silences represent how families connect and communicate. As Anita Vangelisti rightly underscores in her *Handbook of Family Communication*, “If families are created through social interaction, understanding family communication is essential to understanding family members and family relationships” (Vangelisti 2022, p. 1). Moreover, her work showcases how and why family communication is core to family formation and socialization of its members throughout the life course, while also important for ways that families continue or discontinue relations over time, and also the quality of family relations over time (Gottman and Krokoff 1989; Noller and Fitzpatrick 1993; Shimanoff 1980; Vangelisti 2004). Family communication is important for family well-being, not only across time but also across space. Migrant and refugee families represent the quintessential force that bridges migrated families and those who stayed behind. These families must “manage” family health, childrearing, elderly care, and exchange of material and emotional exchanges across borders. This is the core idea underpinning the transnational research turn in migration studies as families now nurture and maintain multiple relationships across borders (Al-Ali 2002; Kibria 2014; Pessar and Mahler 2003).

A Somali proverb that cements the importance of communication in families states, “Nimaan hadlin hooyadiis qadisay”. This proverb links communication to survival for family members. The literal translation of the proverb is, “A man who does not speak up is not even fed by his own mother”. The proverb is often used to showcase the importance of individuals and families articulating what is happening in their lives and sharing their needs so that significant others (immediate and/or extended family members) can come to their rescue and collectively put their efforts into overcoming obstacles.

While communication is essential for refugee and migrant families, its forms are often shaped by the resettlement experiences across the generational and gender axis of power. It is often assumed that those who are either first generation or the 1.5 generation (those who arrived in the US very young) might have different settlement experiences and new modes of communication than their parents and older generations. Bicultural and bilingual younger generations might often straddle between the old culture and new settlement’s dominant culture, with potential chasm emerging from parents whose beliefs and practices might starkly contrast to what the younger generations want and seek to embrace (Bloch and Hirsch 2018; Kibria 1995; McCleary et al. 2020). Differences across generations and the gender divide are most relevant when dealing with cultural practices that were the norm in the old country. At the same time, these might be stigmatized and even illegal in the settlement country.

This research, which utilizes qualitative in-depth interviews with Somali refugee women in the United States, provides an opportunity to dig deeper into refugees’ processing and articulations of their everyday lives, communications, and retrospective reflections of choices made within various settlement contexts within co-ethnics and non-ethnic majority settlements. The experiences of Somali refugees, whose arrival in large numbers in the United States was the result of a devastating civil war that started in the late 1980s, can better help us understand the dynamic nature of cultural changes and continuities as families move across state borders and confront new forms of societal norms and mores that they grapple with in their new homes. Minnesota has the largest Somali refugee settlement in the United States, and understanding this newer community and how families negotiate new and old forms of family dynamics is key to better serving this community in healthcare, education, and other institutions. The questions central to this paper around intergenerational communication also advance our theoretical knowledge of families, gender, and place within migration studies.

In this article, we only focused on women aged 45 and older to explore how they view their communication with younger generations on topics such as FGC and marriage in the context of their new home in Minnesota. We excluded younger women in this paper as most of the communication discussed by those under 45 related to communication about deinfibulation, as we explored in another paper (Connor et al. 2024). While we are not able to ascertain what forces might account for any changes and continuities articulated by our interviewees, we can analyze and interpret how individual women’s narratives grapple with the various cultural contexts lived over their lifetime (e.g., Somalia, Kenya, or Ethiopia as refugee transit countries, and the US as current home). We argue that culture and forms of communication within any society are often dynamic processes that remain malleable. Instead of a deficit model and reification of societies whose cultures are different from “ours” we argue that the “culture” of communication remains adaptable as a settlement in new lands brings about ongoing reevaluation and shifts that both maintain an identity while also remaining open to changes in norms and practices as refugees and migrants encounter new ideas and new forms of knowledge in their transnational connections to families, communities, and institutions.

The narratives presented in this paper illustrate how these women process and articulate, and rearticulate past and present selves, family, larger community input, and voices in decision-making and contact and communication with various institutions such as healthcare systems and healthcare providers. The findings show how Somali women revisit old practices and question certain norms while continuing to maintain core familial, cultural, and religious practices that now align with health-centric norms and futures.

## 2. Method

### 2.1. Participants

Fifteen Somali women aged 45–70 (M = 54.37, SD = 7.85) participated in this study (Table 1). All identified their ethnicity as Somali, their religion as Islam, and were born in Somalia. Women’s education levels varied, with 26% (*n* = 4) having at least some college, 20% (*n* = 3) having a high school diploma, and 53% (*n* = 8) having a lower level of education. Most women (*n* = 9, 60%) worked outside the home full-time or part-time. Almost half (47%) were married at the time of the interview (*n* = 7); 33% (*n* = 5) were divorced; and the remainder were widowed (*n* = 3, 20%). Fifty-three percent had lived in refugee camps before arriving in the U.S. On average, the women had lived in the U.S. for 17.1 years (SD = 7.7; range: 2–26 years). All spoke Somali, and 47% also spoke English (*n* = 7). Most women (*n* = 13, 87%) had type III circumcision.

### 2.2. Procedure

The University of Minnesota Human Research Protection Program reviewed and approved this study, (IRB ID: STUDY00002117) before recruitment. We used convenience and snowball sampling methods to recruit and interview 15 Somali women aged 45 and older in the Minneapolis-St. Paul area during June–October 2019. We intentionally recruited this older group to explore older women’s perspectives on communication about FGC and sexuality with their younger female relatives. This analysis is part of a larger study in which we interviewed 75 women about FGC and sexuality (for more detail, see Chaisson et al. 2023; Connor et al. 2023, 2024; Johnson-Agbakwu et al. 2023). Due to the exploratory nature of this topic (i.e., older women’s perspectives), we chose a sample size appropriate for qualitative interviews (Hennink and Kaiser 2022). The other 60 interviews were conducted with women under the age of 45 and focused more specifically on sexual experiences. Women were approached by bilingual community researchers in community settings (apartment buildings, community centers, community health fairs, Somali malls, etc.) or were referred by another participant.

All interviews were conducted in person at the participant’s preferred location—most (*n* = 14, 93%) chose to meet at home. During scheduling, the interviewers informed the women about the interview process and arranged a meeting location. An in-depth, semi-structured interview allowed for the interviewer to ask probing questions when needed. Interviews ranged from 40 to 102 min (*M* = 58 min, *SD* = 14 min). All interviews were conducted in Somali, per the participants’ choice.

Due to differences between English and Somali, translators did not use word-forword translation but translated words and concepts as close to the original meaning as possible. Somali interviews were translated into English in a two-step process. First, each interviewer/translator verbally translated their interviews using a digital recorder to create English audio files. Second, all translations were back-checked by two people—a bilingual Somali intern and a bilingual Community Advisory Board member—to ensure translation consistency and accuracy. All interviews were transcribed professionally.

### 2.3. Interview Process

At the beginning of each interview, interviewers tried to create a safe and comfortable environment for the women to discuss their experiences by introducing themselves and learning about each woman. As part of the informed consent process, a written consent form (in Somali) was reviewed with the participant. Interviewers gave an overview of the study and purpose, reviewed interview procedures, made sure to allow time for participants to ask questions and clarifications, and demonstrated their understanding of this study before signing the consent form. Women were able to take breaks and pause if needed at any time during the interview. Upon completion, each participant received a USD 75 gift card.

Interviewers kept field notes after each interview, reflecting on observations, interview setting, participant’s perceived comfort and talkativeness, interruptions, distractions, other challenges, relevant pre- or after-interview comments, reactions to the questions, etc.

### 2.4. Materials

The interviewer guide of semi-structured questions and probes was developed based on study aims. Questions covered a range of topics about Somali women who experienced FGC, such as demographics, communication and advice between female family members, family relationships, immigration history, FGC, deinfibulation, mental health, and healthcare experiences. The interview guides differed for the older sample such that questions about sexuality were less personal (per the advice of Somali community researchers), and there was a greater focus on advice-giving. This paper focused on the results of questions about communication and advice-giving related to sex and FGC by the older participants.

### 2.5. Data Analysis

We used a multistage participatory group approach to conduct an inductive analysis of the data centered on Somali women’s voices (Jackson 2008). The analysis team consisted of the first author of Somali heritage, the second author of Somali heritage, and the last author of mixed European heritage. First, the analysis team met via Zoom and read one transcript together. We used open coding as a group to create initial codes in a Word document. Second, the data analysis team jointly used these initial codes to code another transcript; through this process, we agreed upon what codes would be in the preliminary codebook and how to operationalize each code. Third, each analysis team member was assigned three to four transcripts, and the code tree was used to analyze these transcripts. In this stage, any new codes were noted and brought to the larger team for discussion. Fourth, the team reviewed the codes together and collapsed some codes into themes while deleting those that were not salient. For the key themes, a pattern was identified indicating more than a couple participants endorsed the experience. Finally, the first and second authors examined the themes and coded excerpts to generate a cohesive narrative and identify exemplar quotes. We felt confident that the data reached a point of saturation.

## 3. Findings

### 3.1. The Intergenerational Relationship: A Mechanism for Change

Humans do not exist in a bubble. We know that relationships and interactions with others help shape who we are. Relationships are key to realizing where we fit into society, how to behave with people, and understanding society’s implicit and explicit rules. For our Somali participants, intergenerational relationships were essential to acknowledge the impact of migration on family dynamics as well as practices such as marriage and FGC on a personal and communal level. In addition, the transnational characteristics of refugee family systems play an important role in family functioning. Forced migration becomes forced separation. It may be a single child or a grandparent migrating, but the separation of that family member is always salient and sometimes permanent. The existence of an intergenerational relationship and the quality of the relationship played a significant role in the decision-making process of FGC for the younger generation.

### 3.2. Female Intergenerational Relationship Dynamics

Post-resettlement, Somali families had to learn to adjust their family structure after becoming displaced. Families escaped to host countries worldwide, meaning families became separated. This separation of the family members required a shift in structure, authority, and parenting roles. For this reason, participants explained that having an elder in the family system was a privilege. The elders in the Somali community are highly revered as the key holders of cultural norms, community mindset, and family expectations.

Our participant, Munira, a 70-year-old who lived in the U.S. for 15 years, explains the closeness built through travel and phone and video connections with her nieces despite living in another state within the United States:

They call me mom. I’m like their mother. Because they’re my younger sister’s daughters, I helped raise them. They consider me like their mother. If they can’t find their mom or talk to her, they would call me and talk to me. So, I’m like a parent to them [so] they can come to [me] for advice. They also tell their mother that I’m actually a better communicator than her, that I communicate with them better, that I’m more friendly to them than their mother.

The quality of their relationship, not just its existence, was important for Munira *and* her nieces. Munira highlights how she educates them “about our culture, our people, [and] our language. So they like things like that. They like to learn things like that”. She was proud to play such an important role in her nieces’ lives, a role their mother could not fulfill. For Munira and her family, her deep wisdom, knowledge, and experience became an anchor for her family to stay rooted within their culture. Relationships allow for us to ground ourselves within society and build a secure base and a sense of community. Munira was the tether that connected her nieces to a language, a culture, a way of being that was minoritized in greater American society. Through Munira’s memories, experiences, and ancestral wisdom, her nieces can firmly grasp the parts of their identity that are not publicly represented in mainstream society. Munira’s existence in her nieces’ lives protects them from losing community.

Another participant, Idman, who is 56 years old and has lived in the U.S. for three years, described how her niece was practically her daughter:

Yes, I was there for her [niece] since she was born. We were only separated for four years. They came to America before I did, and after I came to America, I was very much involved in her life. I even helped her raise her children… I was always very involved in her life… We look after one another. She’s very helpful to me. She helps me around a lot. She’s like the daughter I never had.

A characteristic of a strong and healthy intergenerational relationship is that it is fulfilling for both individuals. Idman explained that in her relationship with her niece, “we look after one another”. While we highlighted that simply having access to older women in the family system was a privilege, the relationship quality allows for the opportunity to build a secure attachment. The trust cultivated in this relationship becomes one of the main qualifications older women will hold to make executive decisions for the younger generation. However, before discussing decision-making, we want to illustrate what a strong, healthy, connected intergenerational relationship produces: future intergenerational caregiving.

### 3.3. Intergenerational Caregiving

Many of our participants had transnational family systems where some family members lived in other states, countries, or continents. Our participant, Amina, a 61-year-old, recalls the separation of her eldest daughter as she describes her family system.

My eldest daughter and my firstborn, we got separated when the war happened. She was with my relatives, my aunt, so we got separated. She did not come here with me first. She ended up moving to the UK with her grandmother, so she lives there. She lives there now. She’s married. She’s my firstborn. She’s 30 years old.

Amina shared that she raised six children in the US, and four of these were American-born. While Amina was not able to be there for her oldest daughter due to forced separation during the Somali conflict, Amina’s mother became the primary caregiver and parent of the daughter. As common in family separation that occurs with migration, family members most likely stay separated but continue to communicate across international borders. In Amina’s case, her daughter not only lives with and was also raised by her grandmother but was initially with her aunt when the civil war broke out. In contrast to the nuclear family structure of mother, father, and child(ren) unit, this is an example of the expansiveness of the family system and roles that the women within the immediate family realm continue to play in parenting, despite forced migration and despite the family dispersion to distant regions of the globe. To elaborate, the presence of the birth mother does not automatically mean her role is the only female parental role in the family system. Khadijah, a 64-year-old who lived in the US for 22 years, explains how she became the primary caregiver for her grandchildren:

My granddaughter, I’ve had her ever since she was born, and also my grandson, I’ve had him since he was younger. My grandson, his mother did not want to keep him. So, I ended up having to raise him. They’re both the same age. They’re 12. They’re both—they’re each my sons’ children. Both of my sons are their fathers and I’ve helped raise them all.

Intergenerational caregiving is a process that holds the family system together and maintains familial ties despite hardship and separation. Khadijah was a source of support, love, and care not only for one but two generations of grandchildren and great-grandchildren. With trust comes great responsibility and authority. Strong relationships allow for older women in the family system to influence and even make final decisions about important issues regarding their younger-generation female relatives.

### 3.4. Intergenerational Structural Elements in Decision Making

To reiterate, the intergenerational relationship and the presence of older Somali women in the family system allow for the maintenance of cultural norms and the shifting of cultural practices. They become the gatekeepers of the family, the protectors of the young, and the transmitters of culture. They take on roles that many others, including fathers and male clan elders, previously occupied but are no longer viable candidates due to family dispersal and diasporic lives. Isra, a 48-year-old in the US for 22 years, described her role when her younger nieces were thinking about marriage:

My nieces told me before they got married—they told me that they were planning on getting married. I asked them who they were getting married to. I told them that I would not approve if they’re not marrying decent men. I got to know both of my nieces’ husbands before they married them. I made sure that he [they] was [were] from a good family and a good person.

Due to the expansiveness of the Somali family system, the institution of marriage is truly a uniting of two family systems, not just two individuals. While the two people getting married have the final say, evaluating fit, compatibility, and shared understanding happens through the older generation. Our participants shared the same sentiment and explained the importance of family involvement in marriage decision-making. Maryam, a 47-year-old who lived in the US for two years, describes her family’s decision-making process if her daughter moves towards marriage.

No, if my daughter plans on getting married, she’ll tell me first, and I’ll let her dad know about it, and we’ll talk about it and see if the person she is marrying is a good person. If she brings someone who is a good Muslim and prays five times a day, then we’ll support her. We’re not gonna ask for any money. So she’ll let us know if she plans on getting married. She would not hide it from us.

The involvement of the rest of the family system is essential, and each family member plays a particular role. Maryam above showcases both changes in what marriage should entail by her emphasis on not requiring the traditional dowry from the groom but also the continuity of encouraging the groom to be a good Muslim who prays and is a good support for her daughter. These expectations are all forms of socialization within the family microsystem compared to the larger system of society. These intragenerational relationships are the first step in helping the younger generation understand their subsystem’s mores and values. If we think of relationships as the catalyst for developing these strong, grounded ethnic identities, communication is the pathway in which these values become explicit.

While explaining the marriage process, Maryam also inadvertently illustrated the role communication plays, sending messages of expectations during the marriage process to her daughter:

My daughter, if she needs help with any decisions, she comes to me, I give her my advice; she talks to me first before she decides anything… She mentioned she wanted to get married and told me who she was planning on getting married to. I made sure whoever she was marrying was a good person, that he had a good relationship with his parents and family, and also he was somebody who was faithful and prayed five times a day. I also learned about his background from other people Manshallah-God willing.

As a result of the transnational characteristic nature of the family systems of our participants, communication increasingly played an important role in maintaining family functioning despite physical separation. Communication was not only a tool to reinforce family values but a mechanism to instill this knowledge in the next generation. Good communication also serves as a recalibrating tool to establish “normal” family dynamics following the disruptions that displacement and loss of home entail. The explicitness of the communication style is further essential in an environment where these families are minoritized, and younger generations are exposed to other values and expectations of marriage from the dominant culture. This means that communication is not only essential for passing down cultural wisdom and/or knowledge but also shapes perception and understanding in younger generations.

### 3.5. Openness to Challenging Cultural Norms and Communications

Our participants mentioned the importance of open communication and explicitly discussing certain issues and expectations. As previously mentioned, the quality of the relationship often dictates the level and type of communication (openness, intimacy, advice-seeking, etc.) among family members. Participants explained that communicating about vulnerable and taboo topics was easier to initiate and navigate once that secure, safe, and intimate relationship was developed between the older and younger generations. The following section highlights two such topics from our interviews: communication about FGC and sex.

### 3.6. (Non)Communication about FGC

Our participants were honest about their personal experiences with FGC, the impact it had on them, their health complications, and their desire for their younger female relatives not to have the same experiences. For example, Halima, a 53-year-old in the US for 20 years, stated how she communicated with her daughters regarding the type of FGC she chose for them when they were children in Somalia.

I told them why I circumcised them the way I did. I explained to them how it’s not severe or as severe as mine. I explained the reasons. I use an example of one of our neighbors who circumcised her daughter who was around the same age as my nieces. She circumcised her as type three. That little girl faced a lot of health complications.

Communication about FGC was mostly two parts: either (a) the explicit reasoning why type III was not something they could endorse, and/or (b) how, if they were going to follow the practice, the Sunnah type would be the only one they would want for their family members. It is essential for us to highlight that these discussions on FGC were all retrospective, and our interviewees were only reflecting on their now adult children who had FGC either in Somalia, Ethiopia, or Kenya before the family migrated to the United States. Unfortunately for some, their realization of how FGC had such a negative impact on their female family members came after the fact, and they had to live with witnessing the complications that often come with the more severe forms of FGC. Maryam describes her discussion with her eldest daughter about why she came to regret her decision of the FGC type she chose when they were in Somalia:

I always tell her that it was a mistake to circumcise her, and she tells me because she gets very sick, and her experience was really bad, that I should not have circumcises her [*younger*] sisters. I always apologize to my daughter. I tell her I’m sorry, I made a mistake. I tell her that I did not know any better at that time, that I was ignorant.

Maryam, in her conversation with her daughter, acknowledged how her own FCG in rural Somalia also resulted in major health repercussions, including difficulties with urination.

Having explicit conversations about not only the complications of FGC after the fact but also sharing reasons for people who have not had the procedure emerges as equally important. Hearing an elder share why they chose the more severe form of FGC or opted not to continue the practice themselves helps shape the community’s perception as a whole. Discussing FGC outside of the context of regret is just as important. This shows that women who had elders decide not to continue the practice would understand that they are not an anomaly but that cultural and communal understanding is shifting throughout the community settled in various places around the globe.

One participant, Bilan, a 59-year-old living in the US for 23 years, describes how she openly shares her perception of FGC with her family members. She also said she explains to them why she chose not to subject her sister to FGC. Through this discussion, we can see various ways that individual cultural changes have the potential to influence communal mindsets about controversial subjects.

Older people like to circumcise, but not the younger ones. Sometimes, I talk to people back home about it. They ask why I did not circumcise my sister… If I had to circumcise my sister, I would’ve talked to her about it. I would’ve explained to her why it happened and how it happened. I would’ve told her that it was a cultural practice and that we were pressured to circumcise with her. I would have had that conversation with her if I had circumcised her. But I think she’s lucky not to have gone through that.

These messages explicitly sent to the younger generations allow for opportunities to challenge, question, and change old practices. Through this discussion, Bilan’s younger sister is raised in a space where not having gone through FGC is an act of love and protection from her older sister. Moreover, we witness generational shifts about FGC, where grandchildren will soon speak of this practice as one relevant for their grandparents’ generation, and that becomes further and further distant from new American-born first and second-generation Somali Americans. For recent migrants, however, FGC is still closely related to female intimacy, and some of these are tied to conversations about marriage, sex, and female sexuality.

### 3.7. Perceptions about Sexual Communication and Cultural Norms

The fact that sex and FGC are taboo topics in Somali culture partly explains the persistence and prevalence of the FGC practice. When an issue is ignored, kept silent about, and brushed aside, inaction persists. Communication about sex outside of the context of FGC is already discouraged. When FGC discussions are included, the topic becomes even more untouchable. For our participants, anecdotal evidence and reasoning were especially important in showing the younger generations the importance of having these difficult conversations. Many of our participants went out of their way to share their stories and attempted to demystify Somali women’s experiences around sex and sexuality. As Halima shared:

My niece was very curious about my previous marriage. She heard the reason why I left my ex-husband was because I was afraid of sex. A lot of people in my family and community spread rumors about me, that I was afraid of sex. They said the reason why I left my ex-husband was because I was afraid of having sex. That’s what my niece heard. So, my niece asked me about my marriage. I told her the reason why I left my marriage. I also told her that her circumcision is not that bad. I asked her if she was having a hard time with menstruation and urinating. She told me no. I told her then she’ll be okay. I also told her to go see a doctor before she gets married so she can be better prepared. So, she went to the doctor.

Halima was able to change her niece’s experience through open dialogue. Simply sharing her story with her niece and giving her explicit permission to seek professional advice was enough to improve her niece’s experience. We discern how Halima is proud to have given her niece confidence, non-shaming advice, and a completely new outlook on sex, marriage, and health. Unfortunately, this experience is not as common yet, and the majority of our participants still referenced difficulties in opening up to conversations about sex. As such, many Somali women navigate this experience alone, with less support and limited information.

Jawahir, a 57-year-old in the US for seven years, shares one such experience that many Somali women would probably resonate with. Talking about her daughters’ marriages, she stated:

They were virgins before they got married, so there was no need to talk about sex. They knew when they got married it was expected of them to have sex with their husbands. We never talked about it. It’s not culturally appropriate to talk about sex…

Through these two examples, we want to highlight Halima’s bravery in sharing her experience with her niece and how quickly and easily she was able to change her niece’s experience with that simple, open conversation. We juxtapose this interaction with the quote from Jawahir explaining how sex is simply nonexistent in conversations.

Our interviewees also provided a third response that is more general, less anecdotal, and at least acknowledges sex in marriage. Munira provides a good example of what this looks like:

No. It’s [sex] not something we talk about. It’s a cultural thing. We just don’t talk about it. But I did give her advice. I told her to get married [to] somebody she’s happy with, somebody who is a good person and educated, like her. Though we didn’t talk about sex… I would tell her that sex is part of life, is part of human nature, and it’s also a good thing to have in marriage.

Munira explains that while she does not dive into details surrounding sex and intimacy, she does endorse that it is “part of human nature” and “a good thing to have in marriage”. The above narrative emphasizes choosing a “good person” who is compatible and “educated, like her’” These point to strengths in marriages, with potential future sexual issues such as pain that might be addressed with a good partner. This has better chances of leading to good communication and openness to get healthcare intervention when necessary. Here, we glimpse further illustrations of shifts in cultural perception, even at the family and individual levels. The risks these older women are taking by engaging in difficult conversations and being vulnerable by telling their stories are having ripple effects across generations. While subtle, the generalized messages are enough to invite younger Somali women to ask questions and inquire about sex and their sexuality.

### 3.8. Acculturation, Attitudes, and Perceptions: Shifting Tides for Younger Generations

Communicating explicit expectations around culture and tradition allows for transnational and intergenerational Somali family systems to stay connected and integrated. Through our participants, we also know that implicit communication is prevalent in these family systems. With the sentiment of “showing, not telling”, older generations simply refuse to continue FGC with their family members.

Based on their personal experiences, many of our participants did not seek approval before overhauling the practice of FGC in their family systems. This represents acculturation, another critical factor contributing to shifting attitudes and practices. Families and communities that emigrated to the U.S. early on were still in the process of acculturation. This meant they were navigating what practices to preserve and what customs they should take on from the dominant culture—a process of compromising and adjusting.

Ahlam, a 49-year-old living in the US for 25 years, explains the state of the community in its early stages of acculturation:

When we lived in Somalia, girls or women who were not circumcised were insulted or used to be insulted. But when we moved to America, that is when we found out that circumcision is not good for women or girls. People used to say [in the home country] if a girl was not circumcised by the age of six or seven, they would point that out. … I think it causes a lot of damage to women and girls.

The societal pressure to continue a practice in Somalia and neighboring refugee-hosting countries was simply because it was a long-standing practice. The stigma in the eyes of peers and neighborhoods in those contexts produced a form of community policing pressuring all families to maintain the practice. However, through access to advanced medical care in the post-migration US setting, most research participants discovered they could start making decisions about their bodies as adults. They realized that deinfibulation, the opening of a type III FGC, was an option; they did not have to suffer through medical complications (during birth, menstruation, etc.), and it all happened within the confidentiality of the medical system.

Due to their love and care for their family members, and thanks to the confidence these women gain in their conviction that FGC is not a religious or medical necessity, participants now seek to influence family members and communities back home to stop this practice. Halima describes a conversation about this effort:

…Even when I go back home to Somalia to visit my relatives and family, I tell them not to circumcise their daughters. I tell them that God will punish them on the Day of Judgment if they do that to their daughters. I tell my relatives and family back home that they should not cut up their daughters. That’s how God made them. My family back home worries about whether their daughters will be able to marry if they don’t circumcise them. I tell them that I would find good husbands for them who are willing to marry them.

This conversation shows the often conflicting forces at work as to why FGC practice prevails. Men and women who are pushing for the elimination of FGC, such as Halima above, are working against the claims that the practice is necessary for girls to become marriageable in the future. Halima confronts that with her argument that men would marry uncircumcised women. While our research did not include men and their perceptions and support/disapproval of FGC, some research supports Halima’s argument, showing that men often have negative views on FGC when they are confronting the serious medical ramifications of this practice with the healthcare systems in the US (Abathun et al. 2017; Johnson-Agbakwu et al. 2014).

The decision-making power of women such as Halima above might be constrained in Somalia. Still, women have more power and autonomy in the FGC decisions they make in the US context for daughters and nieces or siblings.

One such example was provided by Habiba, a 47-year-old living in the US for 20 years, who explains to us that the health complications were more salient for her, and it was the main reason she did not want her younger sister to be cut. She stated:

I did not talk to my parents about it. I decided that on my own. If I told my parents about it they would’ve told me to circumcise her … I wanted to be open-minded. I did not want to harm my sister.

One way that this practice has remained relevant is that older generations of women assume that Somali men would only marry women who are cut. At the same time, we have participants like Hoodo, who has a strong opinion against FGC and is openly willing to share those with their families and friends, saying, “I think it [circumcision] is a bad thing, and only ignorant people practice it. People did not think it through before they practiced circumcision… And I would like those still practicing it to stop”.

## 4. Discussion

The findings in this study illustrate how these women articulate and rearticulate past and present selves and communicate with family and the larger Somali community. We witness family dynamics and various inputs in decision-making on important but often touchy subjects such as sex, FGC, marriage, and healthcare. The findings show how Somali women revisit old practices and question certain norms while continuing to maintain core familial, cultural, and religious practices that now align with health-centric norms and futures. Multiple studies have documented shifting attitudes towards FGC after migration to western countries, as well as within countries that have a history of the FGC practice. Many of these studies have explored factors outside of the family experience as contributing to shifts, such as legal prohibitions, national campaigns denouncing FGC, and education about the health complications associated with FGC by healthcare professionals (Salah et al. 2024). Our findings demonstrate the need to incorporate the wisdom of female elders into further study of this issue.

The importance of intergenerational relationships cannot be emphasized enough. For Somali family systems, which tend to be transnational, having close relationships with elders becomes a lifeline. Our participants illustrated the beauty and privilege of having access to elders. FGC is a practice that permeates essential aspects of life, including physical health, marriage, intimacy, relationships, sex, and communication (Andro et al. 2014; Connor et al. 2023; Lurie et al. 2020; Pérez-López et al. 2020). Our discussions showed that our participants shifted not only the mindset in their family systems but also across communities. While this study showcases the continuity of family connectedness within generations of Somali women, it also shows us the changes that come about with migration and contact with new systems and new communities. Somali women’s access to various understandings of FGC, and their recognition that the practice does not have any religious basis is most likely bolstered by migration and contact with other Muslim communities, as well as recognition of the negative health experiences in a settlement where the practice is not the norm (Gele et al. 2012; Kawous et al. 2022).

The impact of older-generation Somali mothers and grandmothers now advocating for the end of FGC will be felt for generations. While more needs to be done to stop FGC around the globe, the changes these older Somali generations have begun have already been noticed and felt across generations (Barrett et al. 2021; Johnsdotter and Essén 2016). The diaspora communities often have a lot of power with their families, as they often provide the financial resources that those still in conflict zones rely on. The Somali diaspora thus has the power to bring about further changes by sharing information about the serious health ramifications of FGC as well as the fact that this practice is not required by Islam as many Somalis previously believed. They also have the power to demystify discussion around sex, which was normally a taboo topic. Being in the presence of older family members who understand the importance of having these conversations out loud can shift the experience of women from embarrassment and shame to curiosity and learning. While we often blame the older generations for longstanding harmful practices, we should not forget that those same people also have the capacity to influence change at all levels of society. Holding our intergenerational relationships dear can be a recipe for growth.

## 5. Limitations and Future Research

This research study centered the voices of refugee women over age 45 in examining intergenerational communications around family, marriage, sex, and FGC. Our sample represents a group that is often missing in much of the academic literature. However, there are some limitations to this research: The sample size is small, which is fitting for new topics in qualitative research, but future research could explore these themes in a larger group and with other communities to further our understanding. We had intended to recruit dyads to participate in this study, for example, interviewing a woman over 45 and one of her younger relatives in a separate interview. Such juxtaposition of data from two generations of women would have provided more complex findings to inform how we understand intragenerational communication, gender, and migration. Unfortunately, this was not possible in this research, as the communication discussed by younger women mostly focused on deinfibulation, while the older women mostly focused on displacement, parenting shifts, marriage, and FGC. In the larger study, 35% of women referred friends and neighbors, demonstrating trust in the research process, but almost no one referred a relative of a different generation—even when asked. The two main reasons were geographical distance and discomfort about being in the same study with an older/younger relative when the subject under investigation is about sexual health. Future research could benefit from examining the role of intergenerational relationships using techniques that overcome or at least reduce the challenges that we encountered in this project.

## 6. Conclusions

Shortly after we began working on this manuscript, we learned about this Special Issue emphasizing feminist solidarity and social justice. Though participants did not use these words to frame their experiences, woven throughout the narratives, one can see both concepts emerging. Participants expressed solidarity with the generations of women before them, honoring their culture and teaching the youth about it. They also expressed solidarity with the generations after them, wanting future generations to be free of the health complications that FGC often entails on sex, pregnancy, menstruation, and beyond. One may also argue that the absence of scholarship amplifying older women’s voices is itself a social justice issue. As stated above, female elder relatives are often part of the decision to circumcise their younger relatives, which may result in a simplistic conclusion that they are the problem and others are the solution. We acknowledge that there is a lot of work to be done to eliminate the practice of FGC; not all women throughout the globe will share the same concerns that many of our participants did in wanting FGC to end. However, we also believe a simplistic narrative of the older generation as problematic has not allowed for the beauty of the intergenerational relationship to shine (at least in scholarship). Thus, our findings highlight intergenerational relationships as a form of resource that has not been previously explored. We strongly encourage academics, researchers, healthcare providers, and other vested parties to consider how inclusion of female elders from communities that practice FGC may move both scholarship and advocacy forward.

## Figures and Tables

**Table 1.** Participant demographic and FGC background information (*n* = 15): numbers, percentages, means, and standard deviations.

Variable	*n*	%	*M*	*SD*
Age			54.37	7.85
Years in the U.S.			17.1	7.7
Education				
No school	3	20		
Elementary school/some high school	5	33		
High school diploma	3	20		
Some college/college degree	4	26		
Employment status				
Part-time	5	33		
Full-time	4	27		
Homemaker	1	7		
Unemployed/disabled/other	5	33		
Marital status				
Married	7	47		
Divorced	5	33		
Widowed/separated/never married	3	20		
Lived in a refugee camp				
No	7	47		
Yes	8	53		
Language spoken				
Both Somali and English	7	47		
Somali only	8	53		
FGC type				
Type I	1	7		
Type II	0	0		
Type III	13	87		
Type IV	1	7		

## Data Availability

This study was completed using qualitative interviews. Each participant signed a consent format the time of the interview. The consent form did not contain language about data sharing, and therefore it is not appropriate to share even deidentified data. Additionally, because the data is from individuals who are from a small population, if made public, it may be difficult to guard confidentiality. Researchers may contact the corresponding author to request access to a subset of the de-identified data, and the request will be reviewed with the team for appropriateness.
